# A modified hydrogel production protocol to decrease cellular content

**DOI:** 10.1590/acb371005

**Published:** 2022-12-19

**Authors:** Gabriela Catão Diniz Braga, Cristina Pires Camargo, Martin Conrad Harmsen, Aristides Tadeu Correia, Sonia Souza, Marilia Seelaender, Viviane Araujo Nunes, Jeniffer Farias dos Santos, Elida Adalgisa Neri, Iuri Cordeiro Valadão, Luiz Felipe Pinho Moreira, Rolf Gemperli

**Affiliations:** 1 Bachelor. Universidade de São Paulo – Discipline of Plastic Surgery, Microsurgery and Plastic Surgery Laboratory – School of Medicine – São Paulo (SP), Brazil.; 2 PhD. Universidade de São Paulo – Discipline of Plastic Surgery, Microsurgery and Plastic Surgery Laboratory – School of Medicine – São Paulo (SP), Brazil.; 3 PhD. Associate professor. University Medical Center Groningen – Laboratory for Cardiovascular Regenerative Medicine – Department of Pathology and Medical Biology – Hanzeplein 1, Netherlands.; 4 PhD. Universidade de São Paulo – Department of Cardiopneumology – Thoracic Surgery Research Laboratory – Heart Institute of School of Medicine – São Paulo (SP), Brazil.; 5 Bachelor. Universidade de São Paulo – Department of Cardiopneumology – Cardiovascular Surgery and Circulatory Physiopathology Laboratory – School of Medicine – São Paulo (SP), Brazil.; 6 PhD. Associate professor. Universidade de São Paulo – Department of Clinical Surgery – School of Medicine – São Paulo (SP), Brazil.; 7 PhD. Associate professor. Universidade de São Paulo – Department of Biotechnology – School of Arts, Sciences and Humanities – São Paulo (SP), Brazil.; 8 PhD. Universidade de São Paulo – Department of Biotechnology – School of Arts, Sciences and Humanities – São Paulo (SP), Brazil.; 9 PhD. Universidade de São Paulo – Laboratory of Genetics and Molecular Cardiology – Heart Institute – School of Medicine – São Paulo (SP), Brazil.; 10 PhD. Associate professor. Universidade de São Paulo – Department of Cardiopneumology, Cardiovascular Surgery and Circulatory Physiopathology Laboratory – School of Medicine – São Paulo (SP), Brazil.; 11 PhD. Full professor. Universidade de São Paulo – Discipline of Plastic Surgery, Microsurgery and Plastic Surgery Laboratory – School of Medicine – São Paulo (SP) Brazil.

**Keywords:** Regenerative Medicine, Biocompatible Materials, Hydrogels, Skin, Artificial, Cells, Cultured, Fibroblasts

## Abstract

**Purpose:**

To analyze the cytotoxicity and cell in porcine-derived decellularized skin matrix.

**Methods:**

We analyzed the effect of multiple decellularization processes by histological analysis, DNA quantification, and flow cytometry. Subsequently, we analyzed the most appropriate hydrogel concentration to minimize cytotoxicity on fibroblast culture and to maximize cell proliferation.

**Results:**

After the fourth decellularization, the DNA quantification showed the lowest DNA concentration (< 50 ng/mg). Histological analysis showed no cell components in the hydrogel. Moreover, hematoxylin and eosin showed a heterogeneous structure of collagen fibers. The best hydrogel concentration ranged from 3 to 25%, and there was no significant difference between the 24 hours and seven days.

**Conclusions:**

The process of hydrogel production was effective for removing cells and DNA elements. The best hydrogel concentration ranged from 3 to 25%.

## Introduction

Chronic wounds are considered humanistic and economic burden to the society and patients and are growing as a problem due to ageing population^1^. This condition impairs patients’ quality of life, extends hospital length stays and consequently increases public health costs^1^^-^ 6. In addition, the World Health Organization estimates an increase in chronic wounds, mainly because of a longer life expectancy and the concomitant systemic chronic diseases such as cardiovascular, diabetes, and obesity^2^^,^ 3 ^,^ 5.

Amongst several therapeutic strategies, regenerative medicine has shown great potential for clinical practice^7^^-^ 10, restoring tissue/organ function^7^. The new alternatives are based on three pillars: stem cells, growth factors, and scaffolds^11^^-^ 13. The scaffolds provide a cytoskeleton to promote cell-to-cell and cell-to-growth factors interaction and consequently stimulate cell proliferation and differentiation^11^^,^ 13 ^,^ 14.

Amongst several scaffolds (porous, fibrous, and gels), hydrogels mimic extracellular matrix for soft tissue regeneration^13^. In this sense, decellularized skin may reproduce cytoskeleton architecture, including reticular structure, polymeric chains, and high water concentration^15^. These chemical and mechanical characteristics besides to mimic extracellular matrix can easily carry oxygen, nutrients, and soluble factors, increasing cell viability^15^^-^ 17. The process of producing hydrogel can be mechanical or chemical. Literature data show a better production performance in a chemical process, which decellularizes skin, but keeps the cytoskeleton viability^18^^,^ 19. One of the challenges of the chemical hydrogel protocols is the DNA measurement after lyophilization. Occasionally, the DNA/RNA ratio results in a high value, but sometimes it is not related to DNA residuals chemical residuals in the hydrogel production protocol.

Considering this scenario, this study analyzed the production of a porcine decellularized skin-derived hydrogel to produce a biocompatible scaffold for fibroblast proliferation.

## Methods

This study was approved by the Ethics Committee of School of Medicine of Universidade de São Paulo, called National Experimental Animal Council (Conselho Nacional de Controle de Experimentação Animal), whose approval number is 1370/2019. We followed international guidelines for animal laboratory practice.

### Porcine skin harvesting

Two males swine weighing 20-30 kg were anesthetized (*Sus scrofa domesticus* – Linhagem MS60 Empresa Brasileira de Pesquisa Agropecuária – Embrapa). After anesthesia, the animals were placed in dorsal decubitus, and the abdominal wall was washed with chlorhexidine (Riohex^®^, Rioquímica, Brazil). Then, we resected total abdominal skin with n-15 scalpel. The skin was transferred to sterile recipients containing 0.9% NaCl solution.

### Hydrogel production

The skin was cut in pieces smaller than 1 cm^3^ and triturated in a blender, adding deionized water (dH_2_O) and dry ice until forming pieces smaller than 1 mm^3^. Five mL of triturated skin was added in 50-mL falcons, washed with 1x phosphate buffered saline (PBS), centrifuged at 3,000 × g, at room temperature for 3 minutes (all following centrifugations were done in these conditions), and the supernatant was discarded. Then, 40-mL 1x PBS was added to sonicate at 15 RMS for 1 minute (Fisher F60 Sonic Dismembrator, Fisher Scientific, United States of America). Following, the tubes were centrifuged, washed with 1x PBS, centrifuged, supernatant was discarded and incubated with 35 mL of 0,05% trypsin at room temperature for 3 hours and frozen for at least 24 hours.

The skin was defrosted at room temperature, washed with dH_2_O and 1% antibiotic and incubated in a shaker for 3 hours. Successive incubations of 24 hours in a shaker at room temperature were done with 35 mL of reagents in the following order: saturated NaCl, 1% sodium dodecyl sulfate (SDS), 1% triton X-100, 1% sodium deoxycholate (SD), 21 μL DNase (in 7-mL 1.3 mM MgSO_4_ and 2-mM CaCl_2_) and six washes of 1 hour each with 70% ethanol. Three washes were done between incubation with NaCl, DNase and 70% ethanol and ten or more washes were done after incubation with SDS, triton and SD (the wash process consists in: centrifugation, supernatant discarded, dH_2_O addition, 3 minutes in shaker, centrifugation, supernatant discarded).

**Figure 1:**
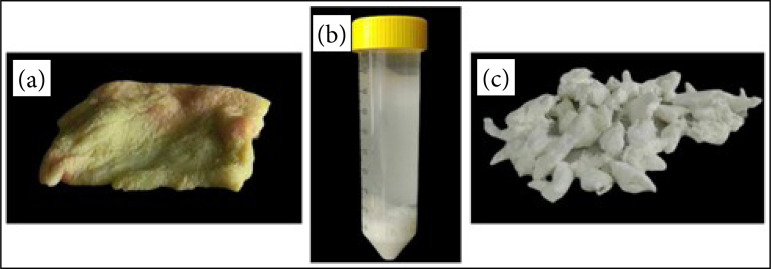
Porcine skin decellularization. **(a)** Porcine skin *in natura*. **(b)** Porcine skin triturated and incubated with reagents (trisol) during the decellularization process. **(c)** Decellularized and lyophilized extracellular matrix (n = 5).

The decellularized matrix was stored overnight with 1x PBS with 2% antibiotic at 4 °C and then lyophilized for 48 hours until totally dry. The decellularized and lyophilized extracellular matrix was stored at room temperature. Four decellularization of porcine skin had been done in different times. The skins used in the first and fourth decellularization are from the same pig, and the initial steps were done once (trituration, washing and freezing), but it was not sonicated for the first decellularization, but for the fourth one after being thawed. The skins used for the second and third decellularization are from two different pigs and had passed through all steps, as written in the given protocol (Fig. 1).

**Figure 2:**
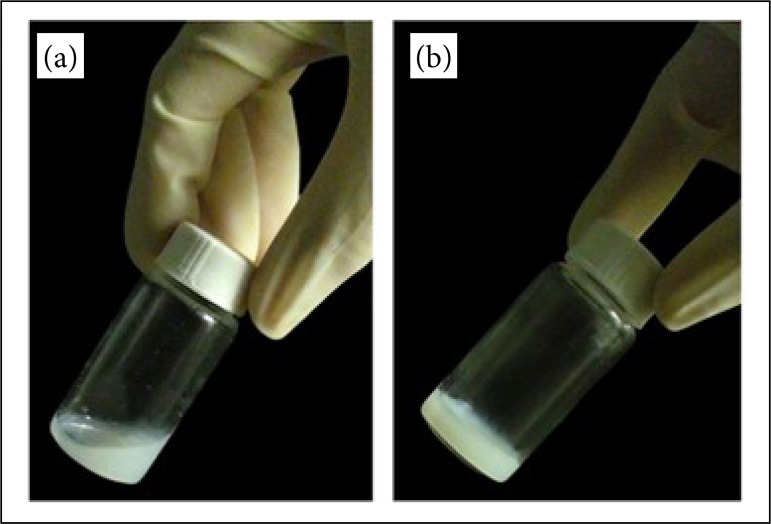
Hydrogel aspect from decellularized porcine skin. **(a)** Hydrogel at 4 °C in liquid state used in the experiments. **(b)** Hydrogel at 37 °C in a swollen state and used in cell culture experiments (n = 5).

For hydrogel production, in a glass tube, 0.2002 g of decellularized extracellular matrix, 0.0204 g of pepsin, 10 mL of 0.01 M HCl, 1% antibiotic and a magnetic bar were added. The glass tube was put in a magnetic stirrer for 24 hours. Then, the hydrogel content was put in a 15-mL centrifuge tube, and the total volume and pH were neutralized with 10% of 0.1 M NaOH and 10% of 10x PBS (% calculated from the total volume). The tube was manually agitated and then centrifuged at 3,800 × g, at 4 °C for 10 minutes. The hydrogel was stored at 4 °C for three months (Fig. 2).

### Hydrogel histological analysis

Two groups were evaluated: hydrogel made by *in natura* porcine skin (control), and decellularized porcine skin, both lyophilized, to evaluate by hematoxylin and eosin (HE) staining the presence of cells in the hydrogel after the protocol for decellularization. To compare the collagen fibers distribution and morphology between the two samples, it was used picrosirius staining. In both protocols, the hydrogel was spread on a glass blade and fixed with formaldehyde 4% for 24 hours.

For typical HE staining, the fixed hydrogel blades were immersed into hematoxylin solution for 3-5 minutes and washed with dH_2_O three times. Then, they were immersed into eosin solution for maximum 3 minutes and washed with different concentrations of ethanol (70, 80, 90, 95, and 100%) and xylene. For picrosirius staining, after dH_2_O wash, the slide was immersed into Sirius red working solution for 1 hour before the steps of alcoholic dehydration. HE analysis was quantitative, and the cell nuclei were counted in ten distinct fields of three glass blades of each group. The microscope used was Labophot (Nikon, United States of America).

### DNA quantification in extracellular matrix

Samples in triplicate containing 10-15 mg of lyophilized porcine extracellular matrix were digested at 55 °C overnight with 5,35 μL proteinase K (18,7 mg/mL) in buffer solution (6.25-mL 6 M NaCl, 25-mL 0.5M EDTA pH 8, and 18.75-mL Milli-Q water). Then, 222 μL of saturated 6-M NaCl and 777-μL chloroform were added, manually homogenized and incubated in a shaker for 1 hour. The samples were centrifuged at 2,500 × g, at 4 °C for 4 minutes, 500 μL of DNA in the supernatant was transferred to a new tube, and 600 μL of cold 100% ethanol was added. The tubes were mixed and centrifuged at 13,000 RPM, at 4 °C for 15 minutes. Supernatant was discarded, 1 mL of 70% ethanol was added and centrifuged in the same conditions. The supernatant was discarded, and the opened tubes were incubated at room temperature for 60 minutes. The DNA was diluted in 100 μL of TE buffer (10mM Tris Base + 0.1mM EDTA pH 8.0), and the samples were heated at 55 °C for 5 minutes in order to dissolve DNA pellets. The samples were vortexed, and 2 μl of them were quantified at the spectrophotometer NanoDrop.

### Fibroblast culture

The fibroblast lineage used was from a 10-month-old human foreskin obtained from Banco de Células do Rio de Janeiro (code nh-skp-FB0050). The cells were cultivated with Dulbecco’s modified eagle medium (DMEM) with 10% fetal bovine serum (FBS) and the antibiotics penicillin 100,000 U/L and streptomycin 100 mg/L, at 37 °C with 5% CO_2_. The culture medium was changed every three days. The fibroblasts were used in the 11^th^ passage.

### Flow cytometry

Fibroblasts were labeled with fluorophore-conjugated antibodies (1:100) against cell markers including positive markers CD29, CD70, CD90, and CD105^20^, and negative markers CD31, and CD45^20^^,^ 21. Propidium iodide (PI) was used to evaluate cell viability. The cells were collected by trypsinization, counted in the flow cytometer (1x10^5^) and centrifuged at 400 × g for 5 minutes at room temperature (all centrifugations occurred in these conditions). Then, a blocking solution (PBS + 2% FBS) was added and incubated at room temperature for 15 minutes. The cells were divided in seven different tubes containing 1x10^5^ cells each. Then, they were centrifuged, the supernatant was discarded, the pellet was resuspended in the spare blocking solution, 1 uL of the antibody was added in each tube, and the cells were incubated for 60 minutes at dark room temperature. Following, the cells were washed with blocking solution and centrifuged twice. Supernatant was discarded. They were resuspended in PBS, and the analysis was done using the software Guava easyCyte in the Guava easyCyte cytometry. The data acquired were referred to 10,000 events and are represented graphically by histogram in logarithmic scale.

### Neutral red uptake indirect cytotoxicity assay

**Figure 3:**
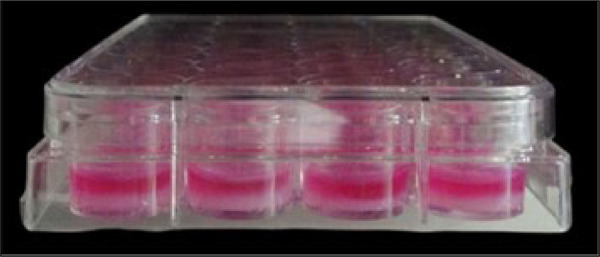
Conditioned culture medium with hydrogel. The content of two phases in the wells are: culture Dulbecco’s modified eagle medium (pink on the surface), and solid hydrogel (white on the bottom) (n = 5).

The aim of cytotoxicity assay of hydrogel was to evaluate whether hydrogel is a possible scaffold to be used in cell culture. The evaluation was made indirectly, which consisted of incubating cells with conditioned culture medium with hydrogel. In order to produce conditioned culture medium, 1 mL of DMEM was incubated with 1 mL of hydrogel at 37 °C for 24 hours and seven days and then stored at -20 °C. The neutral red uptake assay was done according to Repetto *et al*.^22^ (Fig. 3).

### Tridimensional cell culture in hydrogel and staining

Forty μL of hydrogel were added to the 96-well plate, and, after solidification, 100 μL of DMEM was added and incubated overnight. Then, DMEM was discarded, and 1 × 10^4^ cells in 50 μL of DMEM were added to the hydrogel. After 6 hours, fibroblasts were labeled with the cytoplasm marker Calcein AM (405 nm), diluted in DMEM (1:500), and the nucleus marker Hoechst 33342 (360 nm) was diluted in DMEM (1:500). Then, the cells were incubated at 37 °C for 30 minutes. The fluorescence was visualized in the EVOSM7000 Imaging System through the software M7000. The images were processed and rendered in ImageJ.

### Statistical analysis

Interferential analysis of variables was described by mean and standard deviation. The inferential analysis was performed according to the nature and distribution of variables. We analyzed the data regarding media, median, Skewness, and Kurtosis. Then, we plot the information in a histogram and Q-plot graphic. In this case, we chose non-parametric tests (Kruskal-Wallis’ test) and Dunn’s test for post hoc test due to the small sample size.

Alpha p = 5% and study power = 80% were considered. The statistical program used was STATA v14 (StataCorp LP, United States of America).

## Results

### Hydrogel histological analysis

**Figure 4:**
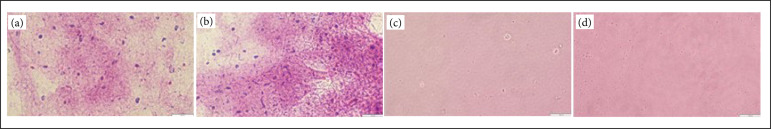
Hydrogel stained with hematoxylin and eosin. (**a** and **b**) Hydrogel from *in-natura* skin with cellular nuclei stained in purple, and (**c** and **d**) hydrogel from decellularized skin without cellular nuclei.

**Figure 5:**
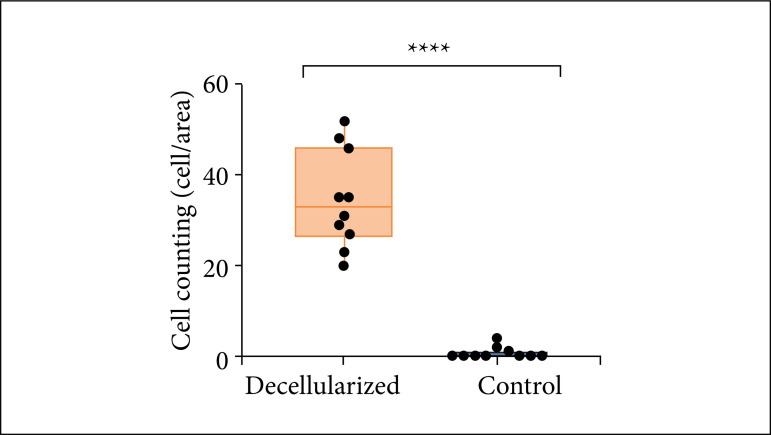
Cell counting in a decellularized hydrogel graph. Control sample mean: 34,6 cells/area. Decellularized sample mean: 0,7 cell/area.

This analysis showed no nuclear elements (Fig. 4) and less cell counting after decellurazation process (Fig. 5).

Magnification of 40x. Scale bar: 500 μm (n = 5).

p < 0.0001 (n = 5).

**Figure 6:**
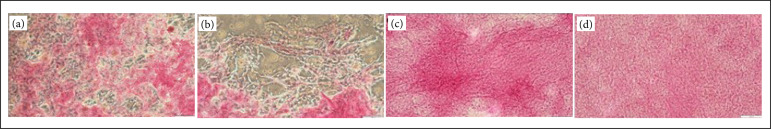
Hydrogel stained with picrosirius. (**a** and **b**) Hydrogel from *in-natura* skin with thicker and more condensed collagen fibers in comparison to hydrogel from decellularized skin, which (**c** and **d**) contain more spread collagen fibers.

The results showed a different pattern of collagen fibers connections between control and decellularized samples (Fig. 6). The hydrogel *in natura* skin resulted in more condensed and thicker reticular connections with more space between collagen fibers. The hydrogel from decellularized skin resulted in three main characteristics:

- The light areas probably are pores between the collagen fibers;
- The dark pink areas may be extracellular matrix not completely digested by pepsin;
- The soft pink areas may represent the extracellular matrix digested by pepsin.

In the hydrogel produced with *in natura* porcine skin (control), there are cellular nuclei (purple), whereas in the hydrogel produced with decellularized porcine skin there is not or there is at maximum one cellular nucleus per area. The comparison of the number of cells between control and decellularized samples has resulted in significant statistical difference (p < 0.0001) (Fig. 6).

Magnification of 20x. Scale bar: 500 μm (n = 5).

### DNA quantification in extracellular matrix

The DNA concentration in the decellularized extracellular matrix resulted in statistical differences between the control and decellularized groups in the second (p = 0.0122), third (p = 0.0126) and fourth (p = 0.0126) decellularization, but there was not significant statistical difference in the first decellularization. Among the decellularization, the only one below 50 ng/mg was the fourth decellularization, with mean of 41,06 ng/mL. Fifty ng/mg is considered the maximum concentration of DNA to not generate immunogenicity in the patient^23^.

**Figure 7:**
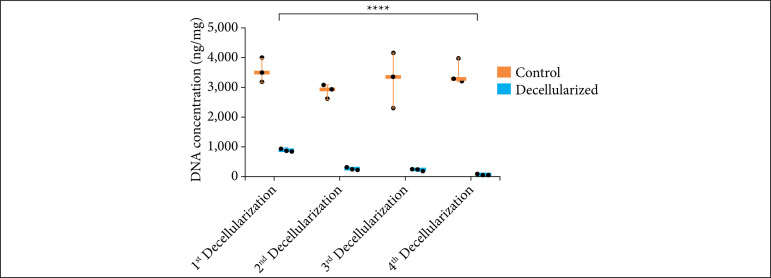
DNA quantification in decellularized porcine skin. First decellularization: control sample: mean = 3,549.67; decellularized sample: mean = 795.821; p > 0.05. Second decellularization: control sample: mean = 2,872.18, decellularized sample: mean = 223.761; p = 0.0122. Third decellularization: control sample: mean = 3,267.7, decellularized sample: mean = 232.3; p = 0.0126. Fourth decellularization: control sample: mean = 3,491.72, decellularized sample: mean = 41.06; p = 0.0126. P compared between all decellularization: first vs. second decellularization: p = 0.001; first vs. third decellularization: p = 0.0007; first vs. fourth decellularization: p < 0.0001; second vs. third decellularization: p > 0.05; second vs. fourt decellularization: p = 0.0008; third vs. fourth decellularization: p = 0.0014 (n = 5).

At the comparison between the decellularizations, there was significant statistical difference between the first and the second (p = 0.001), the first and third (p = 0.0007), the first and the fourth (p < 0.0001), the second and the fourth (p = 0.0008), and the third and the fourth (p = 0.0014) decellularization (Fig. 7).

### Flow cytometry

**Figure 8:**
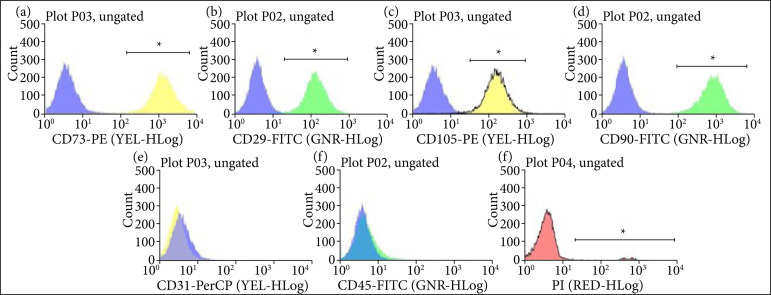
Immunophenotyping of fibroblasts. Results of fibroblast’s immunophenotyping are represented in histogram, with 10,000 events per graph. Cells labeled with antibody anti-human (control) conjugated with fluorophorephycoerythrin (PE) (**a, c,** and **e**) or fluorescein isothiocyanate (FITC) (**b, d,** and **f**) have their population represented at left in the graph. Positive labels for fibroblast: **(a)** anti-CD73 conjugated with PE; **(b)** anti-CD29 conjugated with FITC; **(c)** anti-CD105 conjugated with PE; **(d)** anti-CD90 conjugated with FITC. Negative labels for fibroblast, but positive for endothelial cells: **(e)** CD-31 conjugated with peridinin chlorophyll protein complex (PerCP) and for hematopoietic cells in **(f)** anti-CD45 conjugated with FITC. **(g)** Cells labeled with propidium iodide, marker of dead cells. The left population represents live cells, and the right population represents dead cells (5.03%).

The immunophenotyping (Fig. 8) shows that fibroblasts are positive for the markers anti-CD73, anti-CD29, anti-CD105 e anti-CD90 (Figs. 8a-8d), and negative for the endothelial cells marker anti-CD31 (Fig. 8e) and the hematopoietic cell marker anti-CD45 (Fig. 8f). Therefore, there is no contamination with other cell types in the fibroblast lineage used in this study. According to the dead cell marker, PI, there were only 5.03% dead cells.

### Neutral red uptake indirect cytotoxicity assay

To evaluate the cytotoxicity of hydrogel, the results of serial dilution were compared with positive control (24-hour positive control: mean = 11,054 nm; seven days positive control: mean = 11,652 nm):

- 24-hour serial dilution: in 0% of hydrogel, the cell viability was similar to the positive control; from 3 to 25% cell viability was higher; and in 50 and 100% cell viability was lower;
- Seven days serial dilution: in 0 and 12% of hydrogel, cell viability was similar to the positive control; in 6% cell viability was slightly higher; in 3, 25, 50, and 100% cell viability was lower.

**Figure 9:**
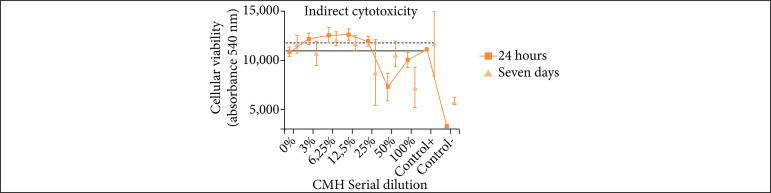
Indirect cytotoxicity with culture medium with hydrogel (CMH) and fibroblasts. The assay was made with a conditioned CMH. CMH was conditioned for two different periods of time: 24 hours and seven days. The serial dilution refers to the percentage of CMH used during cell incubation – 100%: 100-μL CMH; 50%: 50-μL MCH + 50-μL Dulbecco’s modified eagle medium (DMEM); 25%: 25-μL + 75-μL DMEM; 12,5%: 12,5-μL CMH + 87,5-μL DMEM; 6,25%: 6,25-μL MHC + 93,75-μL DMEM; 3%: 3 μL-MHC + 97-μL DMEM; 0%: 100-μL DMEM; Control +: 100-μL DMEM;

For the negative control (24 hours: mean = 3,270.9; seven days: mean = 5,863.39), despite the use of dimethyl sulfoxide (DMSO), cytotoxic solvent, it was seen that there was a cell survivor (Fig. 9). 

Control -: 100 μL dimethyl sulfoxide (DMSO). Dotted lines represent the positive control (n = 5).

### Tridimensional cell culture in hydrogel

**Figure 10:**
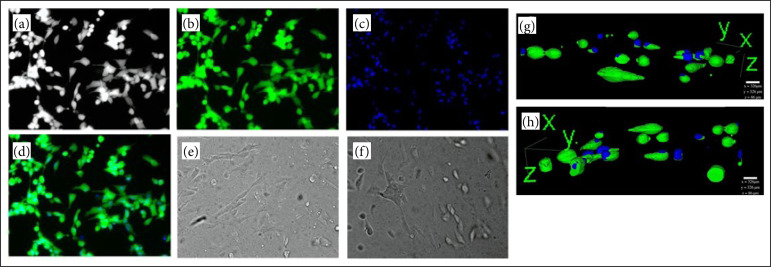
Tridimensional (3D) fibroblast cell culture in hydrogel. The control of this experiment (**a-d**) are fibroblast cultivated in monolayer. **(a)** Fibroblast in grayscale. **(b)** Cytoplasm labeled with Calcein AM. **(c)** Nucleus labeled with Hoechst. **(d)** Overlaid fluorescence of cytoplasm and nucleus. (**e** and **f**) The control of 3D fibroblasts in hydrogel in grayscale. (**g** and **h**) 3D fibroblast in hydrogel labeled with Calcein AM and Hoechst rendered images.

By comparing images from cells in the monolayer of the control group and cells in tridimensional cultivated with hydrogel, we can see that fibroblasts in the hydrogel are in an elongated and fusiform conformation. The images rendered of fibroblasts have also shown the presence of cells in fusiform conformation (Fig. 10).

Scale: x and y = 326 μm; z = 86 μm (n = 5).

## Discussion

The choice of fibroblasts culture for our study is due to their crucial role in wound healing process, mainly because of their characteristics: abundant in the dermis, high proliferation rate, and interaction with other cell types in the wound healing process as mastocytes and keratinocytes^4^^,^ 12 ^,^ 24.

This study showed a successful hydrogel production protocol after four decellularizations. It is crucial to remove all genetic material; genetic material in a hydrogel can increase the chance of rejection to the artificial tissue, and increase the potential of microorganism contamination^23^.

According to Crapo *et al*.^23^, the minimum DNA concentration criteria highlighted is less than 50 ng of DNA per mg of decellularized and lyophilized extracellular matrix (ECM). DNA concentration above this value is directly related to immunogenicity and rejection of the tissue substitute from the host.

In all four decellularization processes done in this study, it was used the same quantity of reagents. When comparing the decellularization of the groups, the biggest difference was between the first and fourth decellularization. The skin used in all groups was from the same animal, and the difference was the use of sonication in the fourth decellularization, whose DNA content was significantly smaller than in the first group (< 50 ng/mg). As the second and third decellularization batches also have passed through sonication and the DNA concentration was about 1/3 lesser than the first, we could suggest that sonication may be essential to the cell removal process. In the fourth decellularization, we also added more dH_2_O washes between the incubations with detergents.

Therefore, the results of DNA concentration decreased with the use of sonication and increased washes between incubations. The best result, under 50 ng/mg DNA concentration, was in the fourth decellularization. For this reason, we only used in the following experiments the fourth decellularization hydrogel. The histological analysis with HE (Fig. 4) has shown that there was an undermost quantity of cells in the decellularized hydrogel, which confirms the low concentration of DNA found in the quantification (Fig. 5).

Despite all washes done and the use of DNase, probably some nucleotides got stuck in the ECM molecules by its chemical affinity, and therefore it is more accurate the use of *Qibit* test to quantify the DNA concentration, since it reads only intact molecules of DNA.

Collagen fibers show to be heterogeneously distributed in all extension of hydrogel. Most parts are properly digested, but few places appear partially digested in all hydrogels obtained from the decellularization process. Similar results were observed by Fernández-Pérez *et al*.^25^.

Macromolecules as collagen remain in the scaffold after decellularization for being large and having intramolecular crosslink^19^, but the collagen fibers could be damaged due to the use of detergent, enzymatic reagents, and sonication. Comparing the collagen fibers in the control and decellularized groups, it is interesting to evaluate with more details the collagen fibers integrity at their mechanical and chemical properties and their functionality as an ECM protein.

It is already known that the use of detergents and enzymatic reagents, in the decellularization process, can be aggressive to the ECM proteins^26^^-^ 28. So, it is possible that these proteins lose their mechanical and chemical properties, leading to a loss of functionality or collagen loss^26^^-^ 28, despite collagens not always being negatively affected^29^.

Morphological analysis showed a heterogenous collagen distribution, resulting in some unfocused areas. Probably, the hydrogel in the blade was also in 3D, which may be an interesting method to analyze how proteins and cells behave inside the scaffold.

Jorgensen’s group discussed the improvement of cell viability and biological and physical properties to develop a bioprinting skin after the addition of decellularized skin ECM (dsECM) in a fibrinogen hydrogel. The results showed that the maintenance in cell viability increased significantly when harvested for 15 days in fibrinogen + dsECM hydrogel in comparison with only fibrinogen hydrogel. Moreover, the addition of dsECM in fibrinogen hydrogel makes it to have a greater structural stability than only fibrinogen hydrogel, and the fibrinogen + dsECM hydrogel harvested with cells after 15 days has shown some results that suggests improvement in the mechanical strength of artificial skin, showing, therefore, the importance of the use of decellularized ECM for artificial skin construction^30^.

In the cytotoxicity results, high difference was not verified between the 24-hour and seven-day tests. The best hydrogel concentration to be used in cell culture with DMEM was 3 and 25% to keep live cells and allow cell proliferation. However, the hydrogel has shown to be cytotoxic in 50 and 100% concentration. We hypothesized that the detergents have not been totally withdrawn during the washes, and the remnant amount is able to kill cells. Fernández-Pérez^25^ has shown that hydrogel from SDS decellularization protocol was not suitable for being used in cell culture due to its cytotoxicity, but hydrogel from triton decellularization was viable for cell culture purposes. Therefore, it is interesting to try a decellularization protocol without SDS.

The tridimensional cell culture in the hydrogel has shown cells morphologically similar to monolayer culture without hydrogel, and, probably, the hydrogel’s microenvironment was viable to cell survival during the first 6 hours. Similar results had been analyzed by Wolf *et al*.^31^, who tested the hydrogel cytotoxicity from decellularized porcine skin in myofibroblasts cultures.

In addition, we hypothesized that the higher cell viability was observed between 3 to 25% of hydrogel, in comparison to control, which may be due to soluble proteins release from hydrogel in DMEM, which interact with fibroblast and work as substrates for cell proliferation, since the DMEM used was FBS free.

## Conclusions

The decellularization was effective for cell lysis, and the DNA concentration was under the threshold that doesn’t generate immunogenicity. The collagen fibers were heterogeneous, with some partial digestion areas, and need more extensive analysis in order to understand their integrity and functionality after being exposed to enzymatic reactions. Taken together, these results suggest that the hydrogel has shown to be cytotoxic depending on its concentration. Nevertheless, the tridimensional cell culture is viable for 6 hours, but it needs to be further analyzed, and the decellularization should be optimized in order to remove the cytotoxicity caused by detergents.

## References

[1] Olsson M, Järbrink K, Divakar U, Bajpai R, Upton Z, Schmidtchen A, Car J. (2019). The humanistic and economic burden of chronic wounds: a systematic review. Wound Repair Regen.

[2] Martinengo L, Olsson M, Bajpai R, Soljak M, Upton Z, Schmidtchen A, Car J, Järbrink K. (2019). Prevalence of chronic wounds in the general population: systematic review and meta-analysis of observational studies. Ann Epidemiol.

[3] Järbrink K, Ni G, Sönnergren H, Schmidtchen A, Pang C, Bajpai R, Car J. (2017). The humanistic and economic burden of chronic wounds: a protocol for a systematic review. Syst Rev.

[4] Zhao R, Liang H, Clarke E, Jackson C, Xue M. (2016). Inflammation in chronic wounds. Int J Mol Sci.

[5] Phillips CJ, Humphreys I, Fletcher J, Harding K, Chamberlain G, Macey S. (2016). Estimating the costs associated with the management of patients with chronic wounds using linked routine data. Int Wound J..

[6] Gould L, Abadir P, Brem H, Carter M, Conner-Kerr T, Davidson J, DiPietro L, Falanga V, Fife C, Gardner S, Grice E, Harmon J, Hazzard WR, High KP, Houghton P, Jacobson N, Kirsner RS, Kovacs EJ, Margolis D, McFarland Horne, Reed MJ, Sullivan DH, Thom S, Tomic-Canic M, Walston J, Whitney JA, Williams J, Zieman S, Schmader K. (2015). Chronic wound repair and healing in older adults: current status and future research. J Am Geriatr Soc.

[7] Mao AS, Mooney DJ. (2015). Regenerative medicine: current therapies and future directions. Proc Natl Acad Sci USA.

[8] Bailey AM, Mendicino M, Au P. (2014). An FDA perspective on preclinical development of cell-based regenerative medicine products. Nat Biotechnol.

[9] Macchiarini P, Jungebluth P, Go T, Asnaghi MA, Rees LE, Cogan TA, Dodson A, Martorell J, Bellini S, Parnigotto PP, Dickinson SC, Hollander AP, Mantero S, Conconi MT, Birchall MA. (2008). Clinical transplantation of a tissue-engineered airway. Lancet.

[10] Mase VJ, Hsu JR, Wolf SE, Wenke JC, Baer DG, Owens J, Badylak SF, Walters TJ. (2010). Clinical application of an acellular biologic scaffold for surgical repair of a large, traumatic quadriceps femoris muscle defect. Orthopedics.

[11] Zhong SP, Zhang YZ, Lim CT. (2010). Tissue scaffolds for skin wound healing and dermal reconstruction. Wiley Interdiscip Rev Nanomed Nanobiotechnol.

[12] Maarof M, Mh Busra, Lokanathan Y, Bt Hj, Rajab NF, Chowdhury SR. (2019). Safety and efficacy of dermal fibroblast conditioned medium (DFCM) fortified collagen hydrogel as acellular 3D skin patch. Drug Deliv Transl Res.

[13] Chaudhari AA, Vig K, Baganizi DR, Sahu R, Dixit S, Dennis V, Singh SR, Pillai SR (2016). Future prospects for scaffolding methods and biomaterials in skin tissue engineering: a review. Int J Mol Sci.

[14] Paschos NK, Brown WE, Eswaramoorthy R, Hu JC, Athanasiou KA. (2015). Advances in tissue engineering through stem cell-based co-culture. J Tissue Eng Regen Med..

[15] Smithmyer ME, Sawicki LA, Kloxin AM. (2014). Hydrogel scaffolds as in vitro models to study fibroblast activation in wound healing and disease. Biomater Sci.

[16] Drury JL, Mooney DJ (2003). Hydrogels for tissue engineering: scaffold design variables and applications. Biomaterials.

[17] Tibbitt MW, Anseth KS (2009). Hydrogels as extracellular matrix mimics for 3D cell culture. Biotechnol Bioeng.

[18] Gilpin A, Yang Y. (2017). Decellularization strategies for regenerative medicine: from processing techniques to applications. Biomed Res Int..

[19] Liguori GR, Liguori TTA, Moraes SR, Sinkunas V, Terlizzi V, van Dongen, Sharma PK, Moreira LFP, Harmsen MC (2020). Molecular and biomechanical clues from cardiac tissue decellularized extracellular matrix drive stromal cell plasticity. Front Bioeng Biotechnol.

[20] Brohem CA, Carvalho CM, Radoski CL, Santi FC, Baptista MC, Swinka BB, Urban CA, Araujo LR, Graf RM, Feferman IH, Lorencini M. (2013). Comparison between fibroblasts and mesenchymal stem cells derived from dermal and adipose tissue. Int J Cosmet Sci.

[21] Lertkiatmongkol P, Liao D, Mei H, Hu Y, Newman PJ (2016). Endothelial functions of platelet/endothelial cell adhesion molecule-1 (CD31). Curr Opin Hematol.

[22] Repetto G, del Peso, Zurita JL. (2008). Neutral red uptake assay for the estimation of cell viability/cytotoxicity. Nat Protoc.

[23] Crapo PM, Gilbert TW, Badylak SF (2011). An overview of tissue and whole organ decellularization processes. Biomaterials.

[24] Kim WS, Park BS, Sung JH, Yang JM, Park SB, Kwak SJ, Park JS. (2007). Wound healing effect of adipose-derived stem cells: a critical role of secretory factors on human dermal fibroblasts. J Dermatol Sci.

[25] Fernández-Pérez J, Ahearne M. (2019). The impact of decellularization methods on extracellular matrix derived hydrogels. Sci Rep..

[26] Syed O, Walters NJ, Day RM, Kim HW, Knowles JC. (2014). Evaluation of decellularization protocols for production of tubular small intestine submucosa scaffolds for use in oesophageal tissue engineering. Acta Biomater.

[27] Partington L, Mordan NJ, Mason C, Knowles JC, Kim HW, Lowdell MW, Birchall MA, Wall IB (2013). Biochemical changes caused by decellularization may compromise mechanical integrity of tracheal scaffolds. Acta Biomater.

[28] Sullivan DC, Mirmalek-Sani SH, Deegan DB, Baptista PM, Aboushwareb T, Atala A, Yoo JJ (2012). Decellularization methods of porcine kidneys for whole organ engineering using a high-throughput system. Biomaterials.

[29] Baiguera S, Del Gaudio, Kuevda E, Gonfiotti A, Bianco A, Macchiarini P. (2014). Dynamic decellularization and cross-linking of rat tracheal matrix. Biomaterials.

[30] Jorgensen AM, Chou Z, Gillispie G, Lee SJ, Yoo JJ, Soker S, Atala A. (2020). Decellularized skin extracellular matrix (dsECM) improves the physical and biological properties of fibrinogen hydrogel for skin bioprinting applications. Nanomaterials (Basel).

[31] Wolf MT, Daly KA, Brennan-Pierce EP, Johnson SA, Carruthers CA, D’Amore A, Nagarkar SP, Velankar SS, Badylak SF. (2012). A hydrogel derived from decellularized dermal extracellular matrix. Biomaterials.

